# Optimization of the Fano Resonance Lineshape Based on Graphene Plasmonic Hexamer in Mid-Infrared Frequencies

**DOI:** 10.3390/nano7090238

**Published:** 2017-08-26

**Authors:** Junbo Ren, Guangqing Wang, Weibin Qiu, Zhili Lin, Houbo Chen, Pingping Qiu, Jia-Xian Wang, Qiang Kan, Jiao-Qing Pan

**Affiliations:** 1 Fujian Key Laboratory of Light Propagation and Transformation, College of Information Science and Engineering, Huaqiao University, Xiamen 361021, China; 1611301026@hqu.edu.cn (J.R.); wgq@hqu.edu.cn (G.W.); zllin@hqu.edu.cm (Z.L.); 1400201017@hqu.edu.cn (H.C.); 1511301022@hqu.edu.cn (P.Q.); wangjx@hqu.edu.cn (J.-X.W.); 2 College of Materials Science and Opto-Electronic Technology, University of Chinese Academy of Sciences, Beijing 100086, China; kanqiang@semi.ac.cn (Q.K.); jqpan@semi.ac.cn (J.-Q.P.); 3 Institute of Semiconductors, Chinese Academy of Sciences, Beijing 100086, China

**Keywords:** graphene oligomer, surface plasmon, Fano resonance, subgroup decomposition, sensing

## Abstract

In this article, the lineshape of Fano-like resonance of graphene plasmonic oligomers is investigated as a function of the parameters of the nanostructures, such as disk size, chemical potential and electron momentum relaxation time in mid-infrared frequencies. Also, the mechanism of the optimization is discussed. Furthermore, the environmental index sensing effect of the proposed structure is revealed, and a figure of merit of 25.58 is achieved with the optimized graphene oligomer. The proposed nanostructure could find applications in the fields of chemical or biochemical sensing.

## 1. Introduction

Plasmonic molecules (PMs), one kind of artificial molecules, are combinations of plasmonic oligomers in nanoscale that can support surface plasmons at specific resonant frequencies, anagously to the process of atoms coupling in chemical molecules to constitute bonds in chemistry [1,2,3]. The sustaining strong charge oscillations result in the enhancement of electromagnetic (EM) fields due to the interaction between the particles. A single nanoparticle in excited plasmonic modes is similar to an isolated atom in electron states. When the nanoparticles are arranged into a cluster, the plasmon modes of the individual nanoparticles interact drastically to constitute the new cluster modes, corresponding to the process that orbitals of individual atomic establish molecular orbitals by linear combination [4]. The analysis of PMs is based on group theory, a math theory widely and deeply applied in molecular chemistry, allowing for a comprehensive description of the structure of PMs [5]. Similar to molecular orbitals, the plasmonic modes are divided into bonding and anti-bonding modes, which depends mainly upon the energy configuration of induced charge and excited field distribution [3]. The exploration for PMs is a study of intense current interest and a good few studies have been conducted recently on the characters of plasmonic dimers [6], plasmonic trimers [7], and more complex structures [8,9] both in theory and applications.

Metallic and dielectric nanostructures can sustain surface plasmon polaritons (SPPs), which provide a wide range of coherent phenomena underlying coupled-oscillator physics, like Fano resonance [10,11,12] and electromagnetically induced transparency [13,14]. Fano resonance first appeared in quantum systems, and has recently become particularly intriguing in the plasmonic field [15,16,17]. The Fano resonance stems from the interaction between strong electromagnetic responses of nanoparticles. Fano resonance or Fano-like resonance is characterized by obvious minima and narrow lineshape in extinction or scattering spectra arising from the coupling and energy translation between the wideband superradiant bright mode and the narrowband subradiant dark mode [18,19,20]. These resonances, caused by the destructive interference, can be explained by plasmon hybridization. However, Fano or Fano-like resonance has another new route to describe that the destructive interference between two neighboring eigenmodes of the subgroups gives rise to the Fano or Fano-like resonance dip [17,21]. Decomposition analysis enables one to modulate and control the overall lineshape by varying the two subgroup modes respectively. Fano resonance is modulated by geometrical arrangement of plasmonic oligomers and nanoparticle parameters such as material, size and shape of the nanoparticles. Owing to the ultranarrow resonance, the Fano resonance possesses potential application in sensing [22]. Various configurations, such as heptamers [11,19,23] and octamers [24,25], have been reported recently. However, on account of the high ohmic dissipation of metal, the traditional plasmonic oligomers based on noble metals are unable to offer Fano or Fano-like resonance with high quality and sensitivity. On the other hand, once the nanostructure of metal oligomers is set up, it is hard to further tune the characteristics of SPPs [26].

Graphene, which is made up of sp^2^ hybridization of carbon atoms, has attracted much attention in recent years due to its unique and prominent behaviors in electronic applications associated with photonics [27,28,29,30,31,32,33]. A monolayer of graphene has two-dimensional carbon atoms arranged in a honeycomb lattice structure. So graphene is usually used for one ultrathin metallic layer, which supports SPPs in terahertz [34,35,36] and mid-infrared frequencies [37,38]. Compared to its counterpart in traditional plasmonic metals, monolayer graphene-supported plasmon possesses the advantages of higher confinement and lower damping loss of the EM field [39]. Moreover, the most attractive property of graphene is the frequency tunability of the plasmons through the change of chemical potential (Fermi energy) [40,41,42,43], whereas the chemical potential is further controllable by chemical doping or external electric field locally [37]. Before the maturation of the fabrication techniques of graphene-based devices, theoretical prediction and numerical simulation are significant [44,45].

In this article, the modulation of Fano-like resonance based on graphene hexamer in mid-infrared frequencies has been adequately discussed and studied by numerical simulations. As a novel material, graphene can support Fano resonances, and possesses prominent frequency tenability [46]. An intuitive method for analyzing Fano-like resonance used in this article is the description of dominant peaks in extinction spectra by separate resonances given by several subgroup modes. The lineshape of Fano-like resonance dominated by subgroup modes in graphene hexamer is optimized as a function of various parameters, such as the size of nanodisk, the chemical potential and the electron momentum relaxation time of graphene. What’s more, compared with PMs consisting of noble metal materials, the higher index sensing effect of the Fano-like resonances in the optimized graphene hexamer is revealed.

## 2. Method

Graphene is a typical two-dimensional material, and is treated as a thin film with a single carbon atom thickness  Δ =0.334 nm. The equivalent permittivity of the graphene monolayer is given by [47] as

(1) $$\varepsilon =1+\frac{i\sigma_{g}{ŋ}_{0}}{k_{0}\Delta },$$

where $\sigma_{g}$ is the complex surface conductivity of graphene, ${ŋ}_{0}\;$= 377 Ohm stands for the impendence of the free space, and $k_{0}$ = $\frac{2\pi }{\lambda }$ is the wave number of the SPP in the air. According to Kubo’s formulation, the complex surface conductivity $\sigma_{g}$ of the graphene monolayer consists of the contributions from both intraband electron-photon scattering $\sigma_{\mathrm{intra}}$ and interband electron-electron transition $\sigma_{\mathrm{inter}}$ [42],

(2) $$\sigma_{g}=\sigma_{\mathrm{intra}}+\sigma_{\mathrm{inter}},\;$$

where

(3) $$\sigma_{\mathrm{intra}}=\frac{2e^{2}k_{B}T}{\pi \hbar^{2}}\cdot \frac{i}{\omega +i\tau^{-1}}\left[\ln\left(2\cos h\left(\frac{\mu_{c}}{k_{B}T}\right)\right)\right],$$

(4) $$\sigma_{\mathrm{inter}}=\frac{e^{2}}{4\hbar }\left[\frac{\sin h\left(\frac{\hbar \omega }{2k_{B}T}\right)}{\cos h\left(\frac{\mu_{c}}{k_{B}T}\right)+\cos h\left(\frac{\hbar \omega }{2k_{B}T}\right)}-\frac{i}{2\pi }\ln\frac{{\left(\hbar \omega +2\mu_{c}\right)}^{2}}{{\left(\hbar \omega -2\mu_{c}\right)}^{2}+{\left(2k_{B}T\right)}^{2}}\right],$$

**Figure 1:**
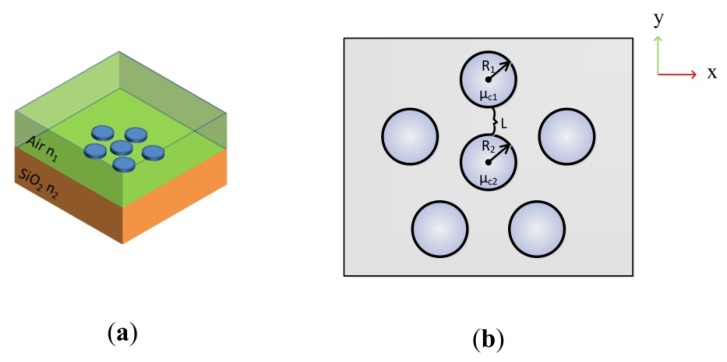
The abridged general view of the graphene hexamer. (**a**) The graphene hexamer lies on the silica substrate with *n*_2_ = 1.5 and is surrounded by air with *n*_1_ = 1; (**b**) The specific parameters of the graphene hexamer.

According to Equations (1)–(4), the complex surface conductivity of monolayer graphene depends on the chemical potential $\mu_{c}$, radian frequency ω and the momentum relaxation time of electron τ. The proposed graphene hexamer structure with D_5h_ symmetry consists of the ring-like pentamer and the central nanodisk as shown in Figure 1. The distance between the outer graphene nanodisks and the central nanodisk is defined as *L*. *R*_1_, *R*_2_ indicate the radius of outer nanodisk and the central nanodisk, respectively. The central graphene nanodisk with a chemical potential $\mu_{c2\;}$ is surrounded by the five outer graphene disks sharing the same chemical potential $\mu_{c1}$. For comparison, *R*_1_, $\mu_{c1}$ and *L* are kept as 60 nm, 0.5 eV, 10 nm respectively in all models. The lineshape of plasmonic resonances is optimized by controlling *R*_2_, $\mu_{c2}$ or τ, which are three crucial parameters for tuning the coupling behavior of SPPs in graphene oligomers. The initial values of *R*_2_, $\mu_{c2}$ and τ are 50 nm, 0.5 eV and 0.5 ps respectively. In reality, the graphene is usually prepared on the substrate like silicon or silica, the refractive index of which is larger than that of air. As shown in Figure 1a, the refractive indices of the air environment *n*_1_ and silica substrate *n*_2_ are set as 1 and 1.5, respectively. 

In our studies, we calculated the extinction cross-section $\sigma_{\mathrm{ext}}$ of graphene hexamer from $\sigma_{\mathrm{ext}}=\sigma_{\mathrm{sc}}+\sigma_{\mathrm{abs}}$, where $\sigma_{\mathrm{sc}}$ corresponds to the scattering cross-section

(5) $$\sigma_{\mathrm{sc}}=\frac{1}{I_{0}}\iint \left(\vec{n}\cdot \vec{S_{sc}}\right)dS,$$

and the absorption cross-section $\sigma_{\mathrm{abs}}$, is given by

(6) $$\sigma_{\mathrm{abs}}=\frac{1}{I_{0}}∭PdV.$$

Here, *I*_0_ represents the incident intensity. $\vec{n}$ corresponds to the normal vector that points outwards from the normal molecule, $\vec{S_{SC}}\;$ is the scattered intensity electromagnetic energy intensity. The integral is taken over the closed surface of the scatter. P indicates the power loss density in the particle, and the integral is taken over its volume.

Using the commercial finite element method (FEM) software, COMSOL Multi-Physics (COMSOL Inc., Stockholm, Sweden), the electro-magnetic properties and spectral response of a symmetric hexamer composed of graphene nanodisks were determined numerically. To provide a comprehensive investigation, a perfect match layer (PML) was set around the model [48]. The proposed structures are illuminated with y-polarized light with normal incident. 

## 3. Results and Discussion

### 3.1. The Extinction Spectrum of the Graphene Hexamer as a Function of R_2_

**Figure 2:**
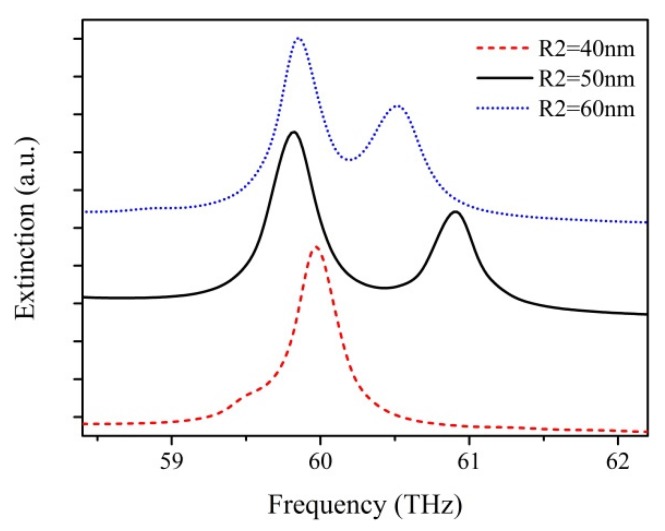
The extinction spectra of graphene hexamer with different radiuses of central nanodisk *R*_2_.

The interaction behavior of SPPs is sensitive to nanoparticle position, size and shape. So these parameters of the nanostructure influence the lineshape of extinction spectra of plasmonic oligomers [15,21,22,24,46,49]. The extinction spectra for graphene hexamers with different central nanodisk radiuses of *R*_2_ = 40 nm, 50 nm, 60 nm were calculated by the above methods, where the distance L between ring nanodisks and the central disk is kept constant at ~10 nm, as shown in Figure 2. For a relatively small radius, say, *R*_2_ = 40 nm, a strong peak obviously appears at 60 THz, but no other peaks show up in the spectrum. This is due to the fact that there is no reaction between the EM fields of the outer ring and the central nanodisk due to long distance. For *R*_2_ = 50 nm, a second peak appears at 61 THz and a Fano-like dip shows up between two resonant peaks. This suggests that the interaction between the EM fields of the outer ring and the central nanodisk have become effective. However, the Fano-like dip is too broad and shallow to be applicable in sensing. When *R*_2_ = 60 nm, the two resonant peaks get closer, which means that the Fano-like dip becomes narrower. For the purposes of better comparison of Fano-like resonance quality, we define the *Q* factor to describe the quality of Fano-like resonance [16]. The *Q* factor is given by *Q* = *f*_0_/δ*f*, where *f*_0_ is the resonance frequency and δ*f* indicates the full width at half-maximum (FWHM) bandwidth of the Fano-like resonance. Nevertheless, the asymmetric line shape of Fano-like resonances leads to an obstacle in defining the FWHM of Fano-like resonances. We use the definition of FHWM of Fano-like resonances as the frequency difference between the Fano-like minima and the peak of the high frequency side of the anti-resonance [50]. The *Q* factors of the Fano-like resonances extracted from the extinction spectra for the different values of the central nanodisk radius *R*_2_ of 50 nm and 60 nm are 123 and 165, respectively. The graphene hexamer with a larger size of the central nanodisk offers higher *Q* factors, which means that the Fano-like resonance is sharper and more applicable for sensing applications. Since the gap between the ring of peripheral disks and the central disk is kept constant, the perturbation of SPP coupling behaviors caused by the distance between ring and central disks is negligible. Only the larger size of the central disk significantly effects the plasmonic coupling between the ring and central disks, therefore creating more new hybridization modes. 

**Figure 3:**
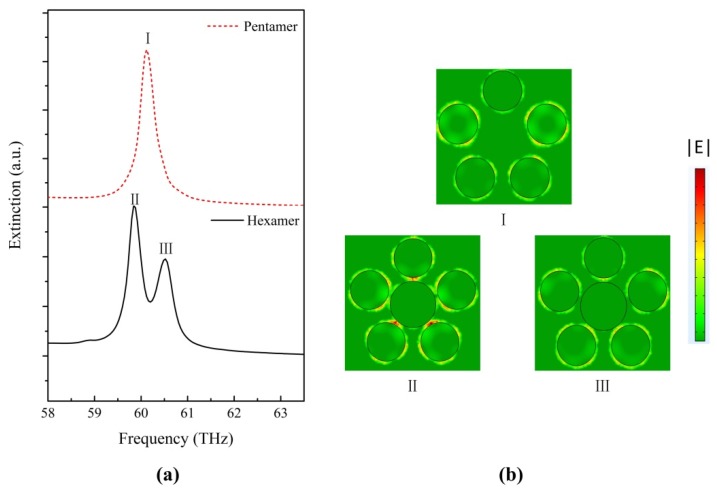
(**a**) Extinction spectra of graphene pentamer and graphene hexamer; (**b**) |E| distribution at the spectral peaks in Figure 3a. The Fano-like resonance is decomposed into two subgroups: the center and the ring.

To further understand the effect of the size of the central nanodisk on plasmonic resonances of graphene hexamer, we calculate the electric field distribution |E| in graphene oligomers at particular spectral positions, as presented in Figure 3. The extinction spectrum of graphene pentamer without a central nanodisk is plotted as a red dashed line in Figure 3a for comparison. In the graphene pentamer, only one plasmonic resonance peak appears at 60.1 THz, and the electric field intensity distribution in the pentamer at peaks (I) is shown in Figure 3b. At position (I), the five disks are efficiently excited, which induces a strong field enhancement around the edge of each disk. We concentrate on the origin of the two plasmonic resonance peaks in the extinction spectra of the graphene hexamer with *R*_2_ = 60 nm. Based on the electric-field intensity distributions in the hexamer at the two peak positions (II) and (III) (shown in Figure 3b), we propose that the two dominant peaks could manifest as a result of two separate resonances arising from two subgroup modes. At position (III), the five peripheral disks show strong electric field intensity around each particle, while the electric field is rarely concentrated on the central nanodisk surrounding. Meanwhile, the central nanodisk is relatively dark. This is a higher energy resonance referred as “ring mode”. The plasmonic coupling behavior of this peak is similar to the peak in graphene pentamer without a central nanodisk. In this mode, the plasmonic coupling mainly occurs on the ring disks and the effect of the central nanodisk is negligible. At position (II), the strongest electric field distribution intensity is concentrated on the gaps between the central disk and the ring disks, and is a lower energy resonance referred to as “central mode”. In this mode, the central disk is no longer isolated, and couples intensively with peripheral disks. The introduction of the central disk presents a route to generate new types of plasmonic resonance. The destructive interaction between the ring modes and central mode is the origin of the formation of the Fano-like resonance. The lineshape of the extinction spectra of graphene hexamer is determined by the ring mode and the central mode. The effect of the subgroup modes illustrates the possibility of modulating the lineshape of Fano-like resonance in graphene hexamer by controlling the two subgroup mode intensities separately. Note that for *R*_2_ = 40 nm, the size of the central graphene nanodisk is not large enough to support central mode, which leads to the absence of Fano-like resonance in the extinction spectrum. This explanation by subgroup decomposition is consistent with the traditional viewpoint that the small size of the central disk gives rise to unmatched cancellation of the whole dipole moment of the peripheral disks and the center disk [22,24]. The reason for there being no alleged Fano-like minimum in the extinction spectrum with a relatively small central disk is that the dipole moment of the ring disks is far greater than that of the central disk in the traditional perspective. Consequently, the geometrical tunability presents the feasibility of modulating the resonance lineshape by subgroup decomposition analyses of Fano-like resonance in graphene hexamer.

### 3.2. The Effect of the Chemical Potential of the Central Disk

**Figure 4:**
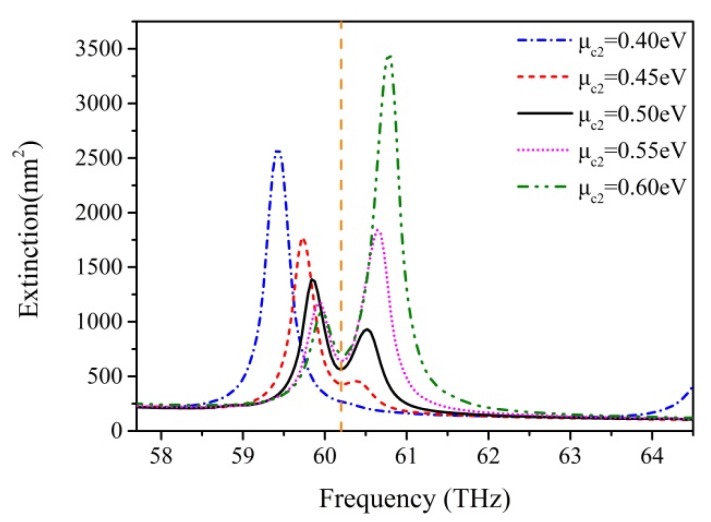
Extinction spectra of graphene with different chemical potentials of the central nanodisk $\mu_{c2}$. The mode competition between central mode and ring mode is clearly visible with varying $\mu_{c2}.$

We change the chemical potential of central nanodisk $\mu_{c2}\;$ and keep the nanodisk size, the distance L and the chemical potential of outer ring disk $\mu_{c1}$ constant. The central disk radius *R*_2_ is set as 60 nm to obtain a high-quality Fano-like resonance. The spectra of variant $\mu_{c2}$ are shown in Figure 4. The variation of the curve is revealed by a systematic change in the relative height of the two peaks, which determine the resonance lineshape of the spectral dip. When $\mu_{c2}$ increases from 0.4 eV to 0.6 eV, the lower energy peak value caused by a central resonance around 59.73 THz becomes lower, which means that the central subgroup becomes darker. The higher energy peak value caused by a ring resonance around 60.59 THz becomes higher, which means that the ring subgroup becomes brighter. The results show an obvious mode competition between central and ring modes by varying $\mu_{c2}$. With a relatively small $\mu_{c2}$, the outer ring energies of SPPs are more inclined to move toward the center, resulting in the enhancement of the central mode; therefore, the ring mode is weak and can even disappear. In contrast, with a larger $\mu_{c2}$, the outer ring energies of SPPs are harder to transmit to the center and concentrate upon the outer ring, resulting in an increase of ring mode and a decrease of central mode. The dramatic competition between two subgroup modes gives rise to a pronounced transformation of extinction dip due to metabolic destructive interference between the two types of resonances Note that the promotion of the total chemical potential of graphene hexamer with an increase in the chemical potential $\mu_{c2}$ of the central disk leads to a shift from low energy to high energy in the extinction spectra. Obviously, the position of a Fano-like dip has little disturbance with variation of $\mu_{c2}$ (vertical dashed line in Figure 4). This lineshape property reveals that graphene oligomers are able to be used in stable Fano resonance sensing applications by tuning the chemical potential of graphene.

**Figure 5:**
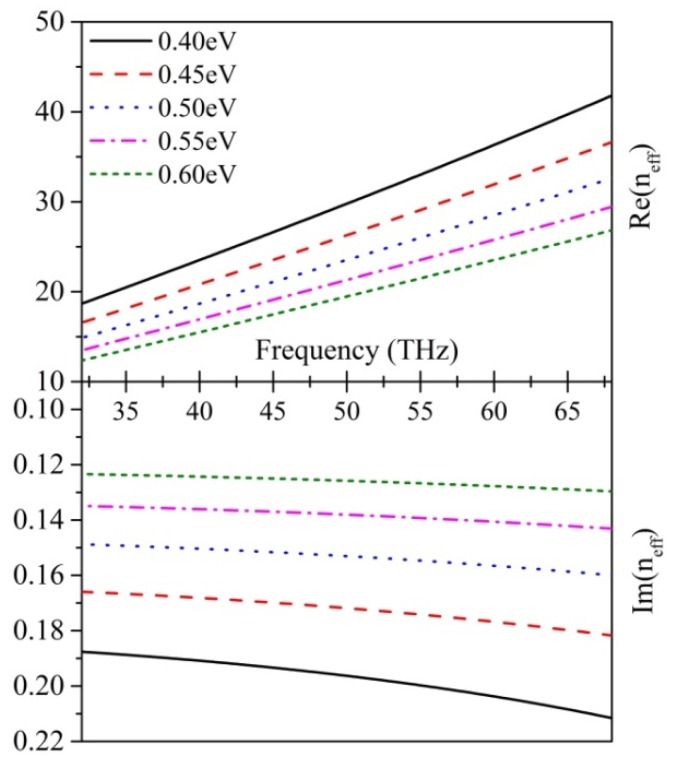
Variation of the graphene effective refractive index in relation to the frequency at various chemical potentials. The top part is the real part of effective index and the bottom part is the imaginary part of effective refractive index.

To shed light more light on the effect of the chemical potential of local graphene on whole graphene oligomers, we calculate the real part and the imaginary part of the effective refractive index *n*_eff_ with different chemical potentials (shown in Figure 5) according to the equation [42]

(7) $$n_{e\mathrm{ff}}=\frac{2i\varepsilon_{\mathrm{eff}}\varepsilon_{0}c}{\sigma_{g}}.$$

$\varepsilon_{\mathrm{eff}}$ represents the effective permittivity of the environment media. It is remarkable that both the real part and the imaginary part of $n_{\mathrm{eff}}$ are inversely proportional to the chemical potential. With an increase in chemical potential, the effects of graphene on confinement and absorption of light become weaker. For graphene hexamer, when adding $\mu_{c2}$, the central graphene disk gradually becomes transparent, which clarifies the reason why ring mode is strengthened by an increase in $\mu_{c2}$. In contrast, when $\mu_{c2}$ decreases, the interaction between the central graphene disk and light becomes more intense, which results in an enhancement of central mode. To ensure a clear observation of Fano-like resonance, we select $\mu_{c2}$ = 0.55 eV, where the two peak values are relatively close and high, to further study the effect on nanosensing.

### 3.3. The Effect of the Momentum Relaxation Time

**Figure 6:**
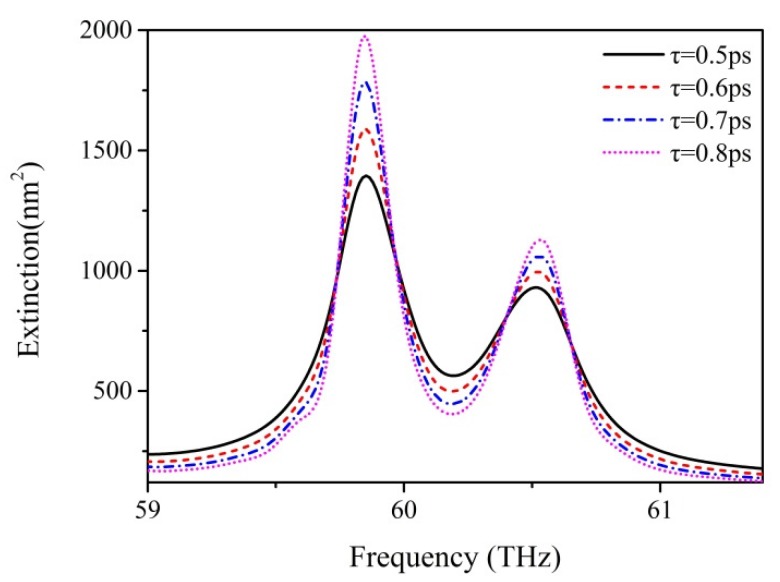
The extinction spectra of graphene hexamer with different momentum relaxation time τ. The two peaks become higher and Fano-like dip becomes deeper with increasing τ.

Further study focuses on the impact of the momentum relaxation time of the graphene nanodisks on the lineshape of Fano-like resonance. The momentum relaxation time of graphene is reduced by dopants, which can increase the scattering of electrons and SPP wave propagation [30]. Additionally, the great impact of momentum relaxation time on graphene is reflected in the transmission performance enhancements of graphene with large values of momentum relaxation time [45]. We select *R*_2_ = 60 nm in order to obtain high-quality Fano-like resonance, and set relaxation times of 0.5, 0.6, 0.7, and 0.8 ps, with the other parameters remaining at their initial values. As shown in Figure 6, a prominent change of the curve shape of extinction spectra is clearly visible. The peaks caused by central mode or ring mode become obviously higher with an increase of τ. The promotion of resonance peaks indicates that the coupling behaviors of both central and ring modes are reinforced. Meanwhile, the dip between the two resonance peaks becomes lower, because the destructive interaction between the ring mode and central mode is also being strengthened. When  τ  reaches 0.8 ps, there is a considerable enhancement of the extinction spectrum, and the robustness of Fano-like resonance is intensified. Remarkably, the amplitudes of peaks and dip change significantly, whereas their frequencies maintain constant values with an increase of τ. On the basis of this observation, there is a feasible route to optimizing the quality of Fano resonance by promoting the relaxation time of graphene. We believe that a relaxation time higher than 0.8 ps would result in a better lineshape of the Fano-like resonance. However, a higher relaxation time strongly relies on the development of the techniques of the material growth and device process. Therefore, we choose a relaxation time of 0.8 ps for further discussion.

### 3.4. Sensing Effect of the Optimized Graphene Plasmonic Oligomer

**Figure 7:**
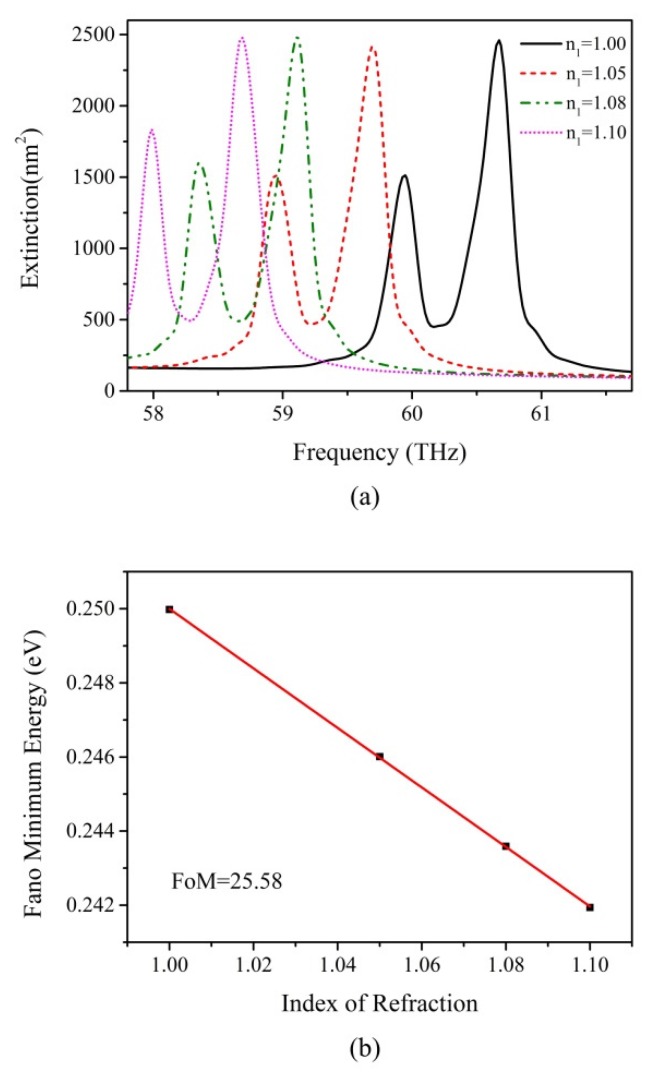
The refractive sensing effect in graphene hexamer. (**a**) The Fano-like resonance has high sensitivity to the variation of environment. It red shifts dramatically with changing the index of refraction n_1_; (**b**) Linear plot of the Fano minimum energy shifts vs refractive index of the surrounding environment. The calculated figure of merit is 25.58.

The optimized Fano-like resonance is narrow and robust, allowing for potential applications such as in refractive index sensors. We investigate the effect of refractive index of an environment n_1_ on extinction spectra with the radius of central disk, the chemical potential of central disk graphene, and the momentum relaxation time of overall graphene kept at 60 nm, 0.55 eV and 0.8 ps, respectively, to ensure the best quality of Fano-like resonance. The results are shown in Figure 7a. The Figure of Merit (FoM) is defined to quantificationally describe the sensing ability of the proposed structure, calculated as the ratio of the plasmon energy shift per environment refractive index n_1_ unit change divided by the width of the spectral peak [51]. To guarantee the rationality of the analysis, we use the definition for the energy of the Fano-like resonance as the midpoint between the energy of the higher frequency peak and the energy of the minimum [22]. To overcome the obstacle of the asymmetric lineshape associated with Fano-like resonances leading to an indefinable FWHM, it is necessary to replace FWHM with a clearer parameter: the width of the spectra. We use the definition of spectral width as the difference of the energies between two adjacent peaks, which has been used to calculate the FoM in previous studies [22,52]. It is calculated as 3.14 × 10^−3^ eV by the average value of spectra with different n_2_. Figure 7b demonstrates the plasmon energy shift as a function of n_1_, and the calculated FoM is 25.58, the value of which is higher than oligomers based on gold and silver, which range from 0.9 to 10.6 [18,22,50,53]. Note that, for available sensing application, we repeat the above simulations with x-polarization direction of incident light, and the results are almost unchanged due to the high symmetry of graphene hexamer, meaning that the graphene hexamer possesses a great deal of flexibility in Fano-like resonance engineering. When it comes to manufacturing a real graphene nanosensor, the optimized graphene hexamer prepared on the SiO_2_ surface of a cleaved fiber with the Focus Ion Beam (FIB) technique is able to realize the nanosensing application.

## 4. Conclusions

In this work, we have shown how the Fano-like resonance in graphene hexamer is modulated by the size of the central disk, the chemical potential of the central nanodisk graphene, and the momentum relaxation time of graphene. The interaction between central and ring modes controls the lineshape of Fano-like resonance. An appropriate increase in the size of the central nanodisk is a practical method to enhance the *Q* factor of Fano-like resonance. More pronounced modulation of the spectral profile is obtained by varying the chemical potential of the central nanodisk in the graphene hexamer. With an increase in chemical potential, the ring resonance becomes more intense, but the central mode exhibits the opposite trend. The increase of momentum relaxation time effectively modifies the robustness of Fano-like resonance in graphene hexamer. On the basis of these optimizations, the FoM of Fano-like resonance in graphene hexamer can reach an extremely high value with unparalleled sensitivity, thereby offering potential applications in chemical or biochemical sensing.
